# Across the ditch: the collective diabetic foot assessment

**DOI:** 10.1186/1757-1146-4-S1-P27

**Published:** 2011-05-20

**Authors:** Belinda Ihaka, Keith Rome

**Affiliations:** 1 Department of Podiatry, Health & Environmental Sciences, AUT University, New Zealand; 2 Health & Rehabilitation Research Institute, Auckland, New Zealand

## Background

The prevalence of diabetes (DM) and its associated manifestations is higher in New Zealand Māori than New Zealand Europeans. There is no current evidence regarding podiatric clinical characteristics of Maori with diabetes. This presentation highlights findings from a group of Maori with diabetes that have foot pathology as determined by a national generic general practice review.

## Methods

Fifty three patients with diabetes were recruited from two Māori Primary Health Organisations. Podiatric specific characteristics, patient demographics and general medical conditions were recorded.

## Results

Fifty three (n=53) Māori patients were recruited for this study with a median age of 54 years (IQR: 45.5 – 61.5 years). We found that Māori had a median duration of diabetes for 15 years, were obese (median body mass index 35.7) with sub-optimal glycaemic control (HbA1c >8% 42%). Podiatric specific characteristics revealed good arterial flow (IQR: Right ankle-brachial index dorsalis pedis 1.1) and a median neuropathy score of 2 (IQR 0, 4). Half the cohort displayed restriction in movement of the right ankle joint (n= 31) and first metatarsophalangeal joints (n=29). Foot education was favourable with positive benefits such as daily foot inspection (n=52). Using a modified classification tool which targets the risk factors collectively, thirty two (60%) patients were identified as requiring regular podiatry management.

## Conclusions

A standardised evidence-based screening and assessment tool could be widely applied by primary care health podiatrists in the detection of imminent diabetes-related foot pathology and to support disease management. This collective assessment would be of particular benefit to communities, including Maori; where the incidence of diabetes and its complications is higher.

